# The incidence of acute myeloid leukemia in Calgary, Alberta, Canada: a retrospective cohort study

**DOI:** 10.1186/s12889-017-4644-6

**Published:** 2017-08-03

**Authors:** Andrea Christine Shysh, Leonard Tu Nguyen, Maggie Guo, Marcus Vaska, Christopher Naugler, Fariborz Rashid-Kolvear

**Affiliations:** 1 Department of Pathology and Laboratory Medicine, Cumming School of Medicine, University of Calgary, Calgary, AB Canada; 2 Calgary Laboratory Services, Calgary, AB Canada; 3 Knowledge Resource Service, Alberta Health Services, Calgary, AB Canada; 4 Department of Family Medicine, Cumming School of Medicine, University of Calgary, Calgary, AB Canada; 5 Diagnostic and Scientific Centre, 2E-415, 9 3535 Research Road NW, Calgary, AB T2L2K8 Canada

**Keywords:** Acute myeloid leukemia, Acute myelogenous leukemia, Cancer epidemiology, Hematological neoplasm, incidence, Canada

## Abstract

**Background:**

The incidence rate of acute myeloid leukemia (AML) was determined in the Calgary Metropolitan Area, a major Canadian city.

**Methods:**

Data from all patients diagnosed with AML between January 1, 2011 and December 31, 2015 were retrieved from a single, centralized cancer cytogenetics laboratory for bone marrow samples, the sole diagnostic facility of its kind in Southern Alberta.

**Results:**

The calculated incidence rate was 2.79 cases per 100,000 person-years with a median age of 60, slightly lower than previously published data. The age-standardized incidence rate for Canada was 3.46 cases per 100,000 person-years. The higher value is reflective of Calgary’s younger population compared to the rest of Canada. Higher male incidence and greatest incidence occurring at approximately the age of 85 is similar to data from other developed countries. The lower incidence rates and median age of diagnosis, in comparison with that of other high-income nations, may be due to differences in the proportion of aging citizens in the population.

**Conclusion:**

This is the first published incidence rate of acute myeloid leukemia (AML) in Canada across all age groups.

## Background

Leukemia is one of the most commonly diagnosed cancers, estimated to have the 11th highest incidence of all cancers worldwide [1]. The incidence of acute myeloid leukemia (AML) is rising in developed countries such as Australia and Canada [2, 3], and incidence in the UK has increased by 73% in the last 40 years [4]. In Canada, the mean age of diagnosis is rising along with AML incidence [2]. As the Canadian population becomes proportionally older, most cancers, including AML, are expected to contribute further to mortality rates [5].

Two systems currently exist to diagnose and classify AML: the French American British (FAB) and the World Health Organization (WHO) classifications. The FAB classification system dates to 1976 and specifies a diagnosis of AML when there is greater than 30% blast population in the peripheral blood and bone marrow [6, 7]. The 2008 WHO revision classifies neoplasms based on morphologic, cytogenetic, clinical, and phenotypic criteria [8]. They define AML as a myeloid neoplasm with greater than 20% blast population in the peripheral blood and bone marrow [7, 9]. The criteria are not absolute; an AML diagnosis can still occur in the absence of reaching the specified blast count threshold if appropriate and specific genetic information is available. The WHO classification defines AML subtypes according to morphological and maturation differences in the blast cells present during diagnosis [9], but also includes specific genetic abnormalities that can be determined by cytogenetics testing. Manifestations of secondary-AML (sAML) and therapy-related-AML (tAML) refer to the development of AML after diagnosis or treatment of a preceding alternate cancer, respectively. Of note, the 20–29% blast range that was formerly considered a myelodysplastic syndrome (MDS) subgroup under the FAB guidelines is revised as AML with multilineage dysplasia according to the WHO [7].

Acquiring accurate and consistent incidence data is vital for long-term AML comparisons worldwide. This is especially relevant as a tool for future health-care resource planning to facilitate appropriate allocation toward patients requiring treatment of specific disease processes. Previous incidence analyses have typically been focused on either youth or adult populations, which have differing incidence and mortality rates. Additionally, a recent study of AML incidence in the United States has raised the issue of possible inaccuracies in previously accepted AML incidence data [10].

This is the first study to provide a population-based incidence rate of AML across all ages in a Canadian population sample. The intention is to establish the incidence of AML in the Calgary Metropolitan Area (CMA) population from recent years (2011–2015) and provide a comparison with corresponding populations worldwide. This will supplement existing global incidence information in order to increase the accuracy of future comparisons and predictions.

## Methods

### Data source

This study was designed as a retrospective cohort. Patient data were obtained from the Calgary Laboratory Services (CLS) Cancer Cytogenetics Laboratory. This facility receives all new bone marrow samples from its catchment area, which includes over 1.8 million residents in Calgary and the surrounding Southern Alberta area. The samples were examined and classified according to the 2008 WHO guidelines via flow cytometry and microscopic hematopathology analysis [8]. The resulting diagnostic reports were evaluated by a cancer cytogeneticist, who ordered the appropriate cytogenetic tests for further analysis. Incident cases of AML were identified for the five-year period of January 1, 2011 until December 31, 2015. Patient postal codes were used to omit patients living outside of the City of Calgary.

### Case identification

New AML cases were categorized by sex and 5-year age cohorts, and incidence rates with 95% confidence intervals were calculated. Calgary population data used in the calculations were taken from Statistics Canada’s Canadian Socio-Economic Information Management System (CANSIM) database in the form of revised and updated population estimates determined by post-censal coverage studies [11]. These estimates provided more accurate measures of population counts than the 2011 census questionnaire, in part by taking into account residents who were missed by the latter. Direct age standardization of the CMA incidence rate to the Canadian 2011–2015 population was calculated using published methods [12] and age-categorized population estimates from a separate CANSIM table [13]. All data analyses and calculations were done in R Studio [14] using R version 3.4.0 [15].

## Results

For the period of 2011–2015, there was an average of 37.8 AML cases diagnosed per year in the CMA, giving a total incidence rate of 2.79 cases per 100,000 person-years, as described in Table 1. The CMA incidence was about 20% lower than the age-standardized rate for Canada, 3.47 cases per 100,000 person-years. The incidence of AML for adults aged 20 and older (3.26 cases per 100,000 person-years) was comparable to those of developed countries worldwide (Table 2). AML incidence rates in world populations generally ranged from 3.0 to 4.0 cases per 100,000 person-years in adult populations, while developing countries had about one-third the incidence of AML.

**Table 1:** Incidence features of Acute Myeloid Leukemia in the Calgary Metropolitan Area (2011–2015)

New cases (per year)	37.8
Crude incidence, all ages (per 100,000 person-years)	2.79
95% C.I.	(2.41–3.21)
Canadian incidence, all ages (per 100,000 person-years)^a^	3.47
95% C.I.	(3.39–3.56)
Crude incidence, ages 20+ (per 100,000 person-years)	3.26
95% C.I.	(2.80–3.79)
Canadian incidence, ages 20+ (per 100,000 person-years)^a^	4.12
95% C.I.	(4.01–4.22)
Male/Female	1.25
Median age of diagnosis	60
Age range	0–95

^a^Age-standardized rate

**Table 2:** Incidence rates per 100,000 person-years, male to female ratio, and median age of diagnosis in select adult global populations

Location	Incidence (per 100,000 person-years)	M/F	Median Age	Reference
Calgary, Canada	2.79	1.25	64	This paper
Canada^a^	3.47	-	-	This paper
Denmark	5.4	1.27	-	Ostgård et al., 2013 [22]
United States	4.1	-	-	SEER^b^, 2016 [30]
United Kingdom	4.0	1.25	68.7	Smith et al., 2011 [31]
Western Australia	3.4	1.40	67	Gangatharan et al., 2013 [32]
SE England (M/F)	(6.24/3.89)	1.53	71	Phekoo et al., 2006 [33]
World^a^	2.2	-	-	Phekoo et al., 2006 [33]
North England	2.68	1.01	63	McGregor et al., 2016 [24]
Brazil	1.11	0.7	42	Capra et al., 2007 [18]
Algeria	0.91	1.16	44.7	Bekadja et al., 2011 [19]
Sweden	-	1.01	71	Hulegårdh et al., 2015 [25]
Ontario, Canada	-	1.15	64	Shabbir et al., 2009 [2]
India	-	-	40	Philip et al., 2015 [20]

^a^Standardized rate from a population subset

^b^SEER: Surveillance, Epidemiology, and End Results

The median age of diagnosis over all ages in the CMA was 60; the median age for adults aged 20 and over was 64. Median ages for the diagnosis of AML in select global populations ranged from the mid- to high-60’s in the UK and western Australia, and a median age of 71 reported in both Sweden and southeast England. The median age of AML incidence in Brazil, India, and Algeria was much lower than that of developed countries, occurring in the low 40’s.

Overall, we found higher incidence in males with a male to female ratio of 1.25 in the CMA. This is comparable to ratios found in many global populations. Exceptions to this male preponderance occurred in Brazil, which had a higher female AML incidence, and equal gender distributions in Sweden and Northern England.

**Fig. 1:**
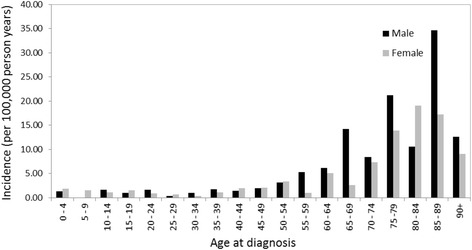
Age and sex-categorized incidence rates of acute myeloid leukemia in the Calgary Metropolitan Area (2011–2015)

AML incidence rates in males and females separated by 5-year age cohorts are summarized in Fig. 1. Incidence rates remained low in early adulthood with gradual increases for both sexes occurring after the age of 35. For females, the incidence rate rapidly increased after the age of 60 to reach peak incidence between ages 80 and 84. For males, the growth in incidence past 65 years is irregular among adjacent age groups. The greatest discrepancies between the sexes are in the high incidence age groups. Specifically, 65–69 and 85–89 year old males had 2–5 times higher incidence compared to females in the same age category and the opposite was observed in the 80–84 year old group. Male incidence peaked at 85–89 year olds, slightly later than for females.

## Discussion

This study is the first to provide population-based incidence data for AML inclusive of all ages in Canada. Other Canadian studies that include AML epidemiological information are limited to youth populations or omit an age-standardized rate [4, 16, 17]. Overall, the incidence of AML in the Calgary metropolitan area is one of the lowest in comparison to reported rates in developed countries. The disparity in incidence rates between international populations may actually be larger since the more strict FAB diagnostic criteria remain in use in certain countries.

The young population in the CMA was expected to result in a relatively lower AML incidence rate and median age since AML incidence usually occurs much later in life. In developing countries with younger populations such as Brazil, India and Algeria, the median ages of AML diagnosis are in the low 40’s [18–20]. The reduced incidence in these countries may be confounded by racial variation since AML is 20% lower in incidence among select ethnic groups compared to non-Hispanic whites [21]. As a neoplasm most common among the elderly, countries with higher proportions of citizens aged 65 and over are expected to have higher incidence of AML. For example, Denmark has a high proportion of senior residents along with the highest reported incidence of AML at 5.4 cases per 100,000 person-years [22].

Although the median age of diagnosis in the CMA is low, the overall age distribution of AML is comparable to that of other countries. AML is commonly known to affect adults in the later stages of life, as seen by the increasing incidence with age, yet the incidence decreases in elderly persons above the age of 85. This may be due to the possible under-diagnosis of AML in the oldest population cohort [23].

The increased incidence of AML in males compared to females is seen in most other populations, although incidence rates in Northern England and Sweden do not show an inclination for either gender [24, 25] and Brazil has higher female incidence [18]. From this study, the gender separation in incidence is not evident until 55–59 year olds, and male predominance is not consistent over all age groups. Male-to-female ratios greater than 1 are widely reported in for most cancer types, especially hematological malignancies, and this may be due to genetic and physiological factors [26].

Gathering population-based incidence data poses several challenges, particularly among developing countries where geographic and economic conditions may be factors. Healthcare models and population composition are among some of the variable properties between regions. The number of healthcare providers in India is relatively low overall but there are higher densities within the central, affluent states [27]. By contrast, the Swedish Adult Acute Leukemia Registry contains information on all diagnosed cases since 1997 with 98% population coverage [28]. The U.S.-based SEER database is often used as a comparative tool for the epidemiology of AML and other cancers, but may not be as accurate as generally assumed [10]. Until 2010, a diagnosis of AML following a previous myeloid malignancy was not registered as AML in the SEER database, possibly decreasing its true incidence [28, 29]. Additionally, algorithm analysis suggests that up to one-third of AML cases were registered in SEER with another disease, often chronic myeloid leukemia or MDS [29]. Such discrepancies in database entry and coding may contribute to the variance in reported incidence rates.

The limitations of this study include the lack of patient data regarding the identification of AML subtypes and mortality rates, which could have provided further insight into the epidemiology of AML. Additionally, the analysis of secondary AML and therapy-related AML may be significant for future epidemiological studies as a link between previous malignant disorders and AML incidence.

## Conclusion

We report a relatively low incidence rate of AML in the Calgary Metropolitan Area, however the age-standardized rate for Canada is comparable to other epidemiological studies. Differences in population dynamics with regards to gender and age distribution may affect the incidence of AML, which has predominance in males and in the elderly. Knowledge of AML epidemiology will aid in the future allocation of healthcare resources as the baby boomer generation enters advanced age.
